# Mothers’ knowledge and attitudes regarding child feeding recommendations, complementary feeding practices and determinants of adequate diet

**DOI:** 10.1186/s40795-020-00393-0

**Published:** 2020-12-01

**Authors:** Kingsley Appiah Bimpong, Ernest Kaa-Emabong Cheyuo, Alhassan Abdul-Mumin, Martin A. Ayanore, Clement K. Kubuga, Victor Mogre

**Affiliations:** 1 Department of Community Health and Family Medicine, School of Medicine and Health Sciences, University for Development Studies, P. O. Box TL 1883, Tamale, Ghana; 2 Department of Paediatrics and Child Health, School of Medicine and Health Sciences, University for Development Studies, P. O. Box TL 1883, Tamale, Ghana; 3 Department of Health Policy Planning and Management, School of Public Health, University of Health and Allied Sciences, Ho, Ghana; 4 Department of Nutritional Sciences, School of Allied Health Sciences, University for Development Studies, P. O. Box TL 1883, Tamale, Ghana; 5 Department of Health Professions Education and Innovative Learning, School of Medicine and Health Sciences, University for Development Studies, P. O. Box TL 1883, Tamale, Ghana

**Keywords:** Knowledge, Attitudes, Child feeding practices, Kpandai, Ghana

## Abstract

**Background:**

Complementary feeding is critical for optimal nutrition in infants and young children as it ensures their growth, health and development to attain their full potential. However, evidence shows that children from developing countries do not meet the core indicators for appropriate complementary feeding. We evaluated mothers’ knowledge and attitudes regarding child feeding recommendations and the determinants of adequate diet among children aged 6–23 months.

**Methods:**

This cross-sectional study included 200 children aged 6–23 months and their mothers/care givers recruited during child welfare clinics of two health facilities in Ghana. Data was collected using a structured questionnaire. Multivariate logistic regression was used to assess determinants of adequate diet.

**Results:**

Sixty-eight percent of the mothers knew the recommended duration of continued breastfeeding, 56.5% how to ensure dietary diversity and enrich their children’s diets and 94% (*n* = 188) had positive attitude towards recommended infant and young child feeding practices. Majority of the mothers (92%, *n* = 183) practiced continued breastfeeding, 10.5% of the children met minimum diet diversity score, 39.5% minimum meal frequency and 8.5% received minimum adequate diet. Determinants of receipt of minimum adequate diet were: having high knowledge in child feeding recommendations (100% vs. 0.0, *p* < 0.001) and child’s father reportedly earning adequate income to cater for the family (AOR = 12.1 (1.32–109.72, *p* = 0.027).

**Conclusion:**

Motherss knowledge levels regarding infant and young child feeding recommendations had notable deficiencies although they generally had a positive attitude towards child feeding recommendations. Knowledge regarding infant and young child feeding recommendations as well as the child’s father having adequate income were important determinants of adequate diet. Nutrition education should emphasize on improving mothers’ nutrition knowledge regarding infant and young child feeding recommendations and supporting mothers to overcome barriers to feed their children with adequate diets.

**Supplementary Information:**

The online version contains supplementary material available at 10.1186/s40795-020-00393-0.

## Background

Child malnutrition is a global emergency affecting every country in the world [1]. Globally, 151 children aged 0–59 months are stunted; 51 million wasted and 38.3 million have excess weight [1]. In sub-Saharan Africa, 39% of children are stunted; 28% wasted and 24% overweight [2]. According to the 2014 Ghana Demographic and Health Survey (GDHS), 19% of Ghanaian children are stunted, 5% wasted, 11% underweight and about 3% are overweight [3]. The Northern Region of Ghana has the highest prevalence of stunting affecting 33% of children aged under 5 years.

A number of underlying factors could be contributing to the rising prevalence of malnutrition in children including poor access to health care, inadequate caring and feeding practices and poor sanitation [4]. Among these, appropriate complementary feeding is very paramount and has been shown to improve nutrition status of children. According to the WHO there are eight core indicators that can be used to assess and guide the feeding practices of young children including 1) early initiation of breastfeeding; (2) exclusive breastfeeding under 6 months; (3) continued breastfeeding for 1 year; (4) the introduction of solid, semi-solid or soft foods; (5) minimum dietary diversity; (6) minimum meal frequency; (7) minimum acceptable diet; and (8) consumption of iron rich or iron fortified foods [5, 6]. Appropriate complementary feeding refers to the timely introduction of solid, semi-solid or soft foods, minimum meal frequency, minimum adequate diet and minimum dietary diversity [5–7]. It has been linked to optimal nutrition in infants and young children as it ensures their growth, health and development to attain their full potential. It’s been shown to reduce child morbidity and mortality thereby increasing child survival and protection [8, 9]. No wonder the WHO recommends that nutritionally adequate and safe complementary feeding should start from age 6 months with continued breastfeeding up to 2 years of age or beyond [8, 9]. Evidence shows that appropriate complementary feeding rates in developing countries are less desirable and majority of infants and young children do not meet the minimum indicators for appropriate complementary feeding [10, 11]. Only 13% of Ghanaian children aged 6–23 months are fed a minimum acceptable diet [3]. Among a sample of 778 children aged 6–23 months from Northern Ghana, Saaka et al. [7] found 57.3% of the children meeting the minimum meal frequency, 35.3% minimum dietary diversity score and 25% had minimum acceptable diet. In Nigeria, Udoh et al. [12] found 31.5% of a sample of 330 children aged 6–23 months from Cross River State meeting minimum diet diversity, 36.5% minimum meal frequency and 7.3% received an acceptable diet. A community-based cross-sectional study among a sample of 506 children from North West Ethiopia found 63% of the children receiving minimum meal frequency, 9.8% for minimum diet diversity and only 8.6% received the minimum acceptable diet [13].

A number of factors have been noted for the less optimal rates of appropriate complementary feeding including maternal education, income levels, antenatal attendance, spouse employment status, quality of institutional healthcare delivery, women empowerment in decision making, and among others [7, 13–15]. Notable among these factors is the mothers’ knowledge and attitudes regarding child feeding recommendations. There is evidence that high knowledge is associated with improved complementary feeding practices among mothers [15–17].

A few studies have investigated adequate diet and its determinants among infant and young children in Ghana. In our search of the literature we only came across three studies [7, 17, 18] that have investigated the core indicators of complementary feeding such as meal frequency, diet diversity, minimum adequate diet and their determinants among children aged 6–23 months. The Saaka et al. [7] study that was conducted in Northern Ghana assessed the three indicators of appropriate complementary feeding but did not assess the knowledge and attitude of the mothers regarding infant and young child feeding recommendations. Gyampoh et al. [17] investigated mothers knowledge regarding recommended infant and young child recommendations and its association with complementary feeding practices but did not assess the mothers/care givers’ attitude towards complementary feeding practices and was also conducted in Accra, which differ socio-economically from Northern Ghana. Frempong and Annim [18] investigated dietary diversity and malnutrition in children using the Ghana Multiple Indicator Cluster Survey for 2011 but did not assess the other indicators of complementary feeding such as meal frequency and acceptable diet. Thus, there is limited data regarding the knowledge and attitudes of mothers on infant and young child feeding recommendations and how these are associated with complementary feeding practices in Northern Ghana and particularly the Kpandai District.

An understanding of the determinants of poor complementary feeding practices within a given context is a necessary step that should be taken to inform the design, planning and implementation of effective and sustainable interventions to improve the nutrition needs of children aged 6–23 months. The current study thus aims to evaluate the knowledge and attitudes of mothers regarding infant and young child feeding recommendations, complementary feeding practices and the determinants of adequate diet among children aged 6–23 months in the Kpandai District of the Northern Region of Ghana.

## Methods

### Study setting, design and participants

This cross-sectional study was conducted during the Child Welfare Clinics (CWCs) of the Kpandai District and the Evangelical Church of Ghana (ECG) Hospitals in Kpandai. Kpandai is the district capital of the Kpandai district. It is located at the South-Eastern Corner of the Northern Region of Ghana. It is bordered to the North by Nanumba South district, East Gonja to the West, Krachi West district to the South-West and Nkwanta North district to the East. The district is largely rural. Agriculture is the main occupation of the majority of inhabitants and has maize, sorghum, millet and yam as staple foods. These are usually harvested from October through December, during which time child care may not be optimal as these rural mothers may lack time due to the harvest. The participants of the study included mothers /care givers with children aged 6–23 months who visited the CWCs of the ECG and Kpandai District Hospitals for growth monitoring and promotion (GMP) services. Mothers/care givers with infants aged 6–23 months of age and were willing to participate were eligible for inclusion. Mothers/care givers with children below 6 months, those with children older than 24 months, those that were sick and those not willing to participate were excluded from the study.

### Recruitment and data collection procedures

Purposive sampling procedure was used to recruit participants. Recruitment and data collection procedures were done by the first (KAB) and second authors (EKC). The hospitals were visited during the study period on days that were scheduled to provide GMP services to mothers/care givers and their children. The mother-child pairs were approached while they waited to receive their GMP service and consent to participate was sought for. Those who agreed to participate were taken through the consent processes, explaining to them the benefits of participating in the study. Voluntary participation was encouraged. Verbal informed consent was obtained from those who could not read nor write in English and written informed consent obtained from those who could read and write in English. In a secluded area at the centre, a paper-based questionnaire was administered face-to face in the local dialect to the mothers who agreed and consented to participate in the study. The questionnaire was completed within 10–20 min. All data collection procedures, methods and informed consent procedures were approved by the Tamale Teaching Hospital Ethical Review Committee.

### Data collection tools

All data was collected using a questionnaire (Additional file 1). The items of the questionnaire were adapted from the Food and Agriculture Organization questionnaires for assessing knowledge, attitudes and practices concerning nutrition and feeding of infants and young children [19]. Components of nutrition knowledge that was assessed were: duration of continued breastfeeding, age of start of complementary feeding, reasons for giving complementary feeds and ways of making complementary feeds more nutritious.

The knowledge scale had 7 items and consisted of both open-ended questions and multiple-choice questions. Each question was scored 1 for a correct answer. Total scores were generated for each participant and computed out of 100%. Components of the attitude towards infant and young child feeding recommendations that was assessed were: confidence in preparing meals, giving a variety of meals, feeding frequency and possible barriers associated with them.

The attitude scale had 7 items that were answered on a 3- point-Likert scale. Two forms of the 3-point Likert scale were used depending on whether the item was assessing perceived barriers or perceived benefits. For perceived barriers the responses were: 1- not difficult, 2- So-so and 3-Difficult. For perceived barriers: 1-Not good, 2-not sure and 3-Good. In order to ensure higher scores denoted positive attitude, items for the perceived barriers were reversed score (i.e. 1 = 3, 2 = 2, 3 = 1). Total scores were generated for each participant and computed out of 100%.

Complementary feeding practices were assessed based on the mother/care givers recall of foods consumed by the child. Key components that were assessed according to the WHO guidelines were continued breastfeeding, meal frequency and diet diversity. Regarding meal frequency, mothers/care givers were asked to indicate the number of times the child ate in the past 24 h. Mothers were asked to indicate whether the child was still breastfeeding (Yes/No response).

Concerning dietary diversity, a list of food items from six food groups were provided and mothers were asked to indicate whether the child had taken any of the foods within a respective food group. This was obtained by summing the number of unique food groups consumed in the last 24 h (FAO, 2011). For instance, if a child was reported to have eaten at least one of the foods listed in a particular food group, the participant was scored 1 for that food group. The food groups were: grains, roots and tubers; dairy products; vitamin A rich foods; flesh foods; eggs; fruits and vegetables; and legumes and nuts. Mothers responses to these questions were used to generate complementary feeding indicators: minimum meal frequency, minimum diet diversity score and minimum adequate diet.

Following the WHO/UNICEF guidelines, a child was considered to have met the minimum meal frequency if in the last 24 h he/she received the minimum frequency for appropriate complementary feeding (i.e. 6–8 months = 2 times; 9–11 months = 3 times, 12–23 months≥3 times; non-breastfed child = 4 times) [5, 6]. According to the WHO/UNICEF and as adopted in this study, minimum dietary diversity refers to the proportion of children aged 6–23 months who received at least a food from at least four of the seven food groups in the last 24 h [5, 6].

A child was considered to have met minimum acceptable diet if he/she had met both minimum meal frequency and minimum dietary diversity. Thus, minimum acceptable diet was the proportion of children aged 6–23 months who met both minimum meal frequency and minimum dietary diversity.

Socio-demographic characteristics such as child’s age, mothers’ level of education, marital status, religion, mother’s employment status, and child’s father having adequate income (This was assessed by the question: Do you think your child’s father or husband earns adequate income to cater for the family: Yes/No.) were also evaluated using the questionnaire. The questionnaire was piloted on a sample of 20 mother-child pairs and the issues that were identified were used to revise the questionnaire to allow for easy understanding and comprehensibility among the participants. In addition, a multidisciplinary team comprising a nutritionist, paediatrician, public health specialist and a behavioural scientist evaluated the final version of the questionnaire for content validity (i.e. relevance, completeness, clarity, and meaningfulness). Suggested revisions were made and the final version approved for data collection. The data from the pilot evaluation were not included into the analysis for the current study.

### Statistical analysis

We analysed the data using the Statistical Package for the Social Sciences (SPSS) software. Descriptive statistics of mean, standard deviation and frequencies were used to describe the data.

The dependent variable was minimum adequate diet which was classified into those who met the criteria (Yes) and those who did not meet (No). Independent variables were child’s age (6–11 months vrs. ≥ 12 months), mothers age (< 30 years, ≥ 30 years), mother’s level of education (No formal education, High, Low), mothers employment status (Employed, Not employed), Child’s father having adequate income (Yes/No), marital status of mother (Married, Single), and religion (Christianity, Islamic, Traditionalist). To evaluate determinants of adequate diet the following analytical approaches were used: univariate and multivariate tests. The Univariate tests adopted were Chi-square test and Fisher’s exact test. Fixer’s exact test was used for responses that were less than 10 participants. To identify factors associated with minimum adequate diet while adjusting for confounders, multivariate logistic regression (a priori selection) was conducted. A *p*-value of less than 0.05 was considered significant.

## Results

In all, 215 mother/care giver-child pairs were approached in which 200 agreed and consented to participate in the study. Table 1 shows the demographic characteristics of the mothers’/care givers and their children. The mean (SD) age of the mothers/care givers was 27 (5.12) years. Majority of the mothers/care givers were married (96%), 49.0% had no formal education and 77.0% were Christians. The mean (SD) age of the children was 12 (5.15) months and the majority were males (56.5%).

**Table 1:** General and social demographic characteristics of the mothers /care givers

Variable	Frequency	Percentage
**Age**		
< 30 years	150	75
≥ 30 years	50	15
**Marital status**		
Married	192	96.0
Not married	8	4.0
**Educational level**		
No education	98	49.0
Low level education	41	20.5
High level education	61	30.5
**Religion**		
Christianity	154	77.0
Muslim	38	19.0
Traditionalist	8	4.0
**Employment Status of the mother**		
Employed	146	73.0
Unemployed	54	27.0
**Child’s father earns adequate income for family upkeep**		
Yes	129	64.5
No	71	35.5

### Knowledge regarding infant and young child feeding recommendations

Table 2 shows mother’s/care giver’s knowledge in infant and young child feeding recommendations. Sixty-eight percent of the mothers/care givers knew the recommended duration for continued breastfeeding. Regarding the recommended age at which a baby should be given complementary foods, 72% rightly said after 6 months. Eighty-four percent of the mothers/care givers knew the right consistency of meal, to be given to children, but only 27.5% were able to give the reason for consistency of meal by reporting that thick porridge is more nutritious because it is prepared with different types of foods or ingredients (food diversity). Eighty-one percent of mothers/care givers had knowledge on ways of encouraging young children to eat. In all, the mothers had a mean (SD) knowledge score of 58.0 (28.45) %. Based on the scores, 52.0% (*n* = 103) of the mothers/care givers had high level of knowledge.

**Table 2:** Proportion of mothers’/care givers’ who had knowledge in infant and young child feeding recommendations

Variable	Frequency (%)
Correctly answered recommended duration of continued breastfeeding	137 (68.5%)
Correctly answered age of start of complementary foods	144 (72.0%)
Gave good reasons for giving complementary foods at 6 months	11 (5.5%)
Correctly knew how to ensure consistency of meal	168 (84.0%)
Gave good reasons why consistency of meal of meal is necessary	55 (27.5%)
Correctly knew how to ensure dietary diversity and ways of enriching porridge	113 (56.5%)
Knew responsive feeding	163 (81.5%)
Mean (SD) knowledge score (%)	58(28.45%)
**Classification of knowledge scores**	
High (> 70%)	104(52.0%)
Low	96(48.0%)

### Mothers’ attitudes towards infant and young child feeding recommendations

Table 3 shows the mothers’ attitudes towards infant and young child feeding recommendations. Ninety-three percent (*n* = 185) of the mothers expressed confidence in preparing food for their children and 51% expressed difficulty in giving different types of food to child. Inadequate finance and inadequate nutritional knowledge were the reasons giving by mothers/care givers for their difficulty. Ninety-three percent knew the perceived benefits of continuing breastfeeding beyond 6 months, with 7.5% expressing difficulty in executing this citing time and work constraints as contributing reasons. Based on the attitude scores, 94% (*n* = 188) had positive attitude towards recommended infant and young child feeding recommendations.

**Table 3:** Mothers’ attitudes towards infant and young child feeding recommendations

Variable	Frequency (%)
Feels confident in preparing food for child	185(92.5%)
Perceives that giving different types of food is beneficial to child	179(89.5%)
Has difficulty giving different types of food to child	103(51.5%)
Perceives that feeding child several times each day is beneficial	171(85.5%)
Has difficulty feeding child several times a day	96(48.0%)
Perceives that its beneficial to continue breastfeeding beyond 6 months	198(99.0%)
Has difficulty continuing to breastfeeding beyond 6 months	15(7.5%)
Mean (SD) attitude score	87.3(12.18%)
**Attitude classification**	
Positive (> 70%)	188(94.0%)
Less Positive	12(6.0%)

### Child complementary feeding practices

Regarding, the consumption of at least one unique food item in the last 24 h from the food groups evaluated, 16.5% (*n* = 33) gave food to their children from the legumes and nuts group; 68.0%(*n* = 136) grains, roots and tubers; 13.5% (*n* = 39) dairy products; 27.0% (*n* = 54) flesh foods; 14.5% (*n* = 29) eggs; 20.5% (*n* = 41) vitamin A rich foods; and 5.0% (*n* = 10) fruits and vegetables. A greater proportion of the mothers (92%, *n* = 183) were currently breastfeeding; 10.5% (*n* = 21) of the children met minimum diet diversity score; 39.5% (*n* = 79) met minimum meal frequency and 8.5% (*n* = 17) met minimum adequate diet.

### Determinants of minimum adequate diet

Table 4 shows the univariate and multivariate determinants of minimum adequate diet. Significant determinants of adequate diet were mothers/care givers having high knowledge in child feeding recommendations; and the father of the child reportedly earning adequate income for the upkeep of the family.

**Table 4:** Univariate and multivariate determinants of minimum adequate diet for complementary feeding

Variable	Univariate			Multivariate		
	Met minimum adequate diet					
	Yes (***n*** = 17)	No (***n*** = 183)	***p***-value	B	AOR (95% CI)	***p***-value
< 30 years	15(88.2%)	135(73.8%)	0.249	−1.43	0.2(0.44–1.29)	0.097
Married	1(5.9%)	7(3.8%)	0.515	−1.30	0.3(0.02–3.99)	0.343
Child’s mother has high level of education	8(47.1%)	53(29.0%)	0.166	0.19	1.22 (0.32–4.67)	0.778
Child’s mother is employed	14(82.4%)	131(71.6%)	0.41	0.46	1.6(0.32–7.84)	0.574
Child’s father earns enough	16(94.2%)	113(61.7%)	0.007	2.49	12.1(1.32–109.72)	0.027
High knowledge level	17(100%)	87(47.5%)	< 0.001	NA	NA	NA
Positive attitude	17(100%)	171(93.4%)	0.604	NA	NA	NA
6–11 months old	12(70.6%)	88(48.1%)	0.097	−0.30	0.7(0.21–2.56)	0.633
Male	7(41.2%)	105(57.4%)	0.213	−0.39	0.7 (0.06–8.29)	0.762
Currently breastfeeding	16(94.1%)	168(91.8%)	1.000	−0.62	0.5(0.06–5.08)	0.588

*NA* Not applicable. All children who met the minimum adequate diet had parents who had high knowledge level and positive attitude

## Discussion

In this study we investigated mothers’ knowledge and attitudes regarding infant and young child feeding recommendations. In addition, we investigated complementary feeding practices and the determinants of adequate diet among children aged 6–24 months.

An important finding of this study was that a greater proportion of the mothers/caregivers (92%) were practicing continued breastfeeding indicative of meeting the infant and young child feeding recommendation for mothers/caregivers to continue to breastfeed as well as giving complementary foods after 6 months. Our finding is similar to the 97.6% reported by Saaka et al. [7] among a sample of children aged 6–23 months from Northern Ghana.

Another important finding of this study was that 39.5% of the children met the minimum meal frequency, 10.5% minimum diet diversity score and 8.5% were fed minimum adequate diet. This is one of the lowest rates of infant and young child feeding practices in Ghana and other developing countries. In a study that consisted of a sample of 778 children aged 6–23 months from three northern regions of Ghana, Saaka et al. [7] found 57.3, 35.6 and 24.9% of the children meeting minimum meal frequency, minimum diet diversity and minimum adequate diet respectively. In rural Madagascar, Rakotonirainy et al. [20] found 50% of a sample of 1824 children aged 6–23 months meeting the minimum adequate diet. Among a sample of children from South West Ethiopia, Edris et al. found 38% of the children attaining minimum diet diversity score [21]. The findings of this study are however comparable to the 8.6% of children aged 6–23 months meeting minimum adequate diet reported among children from a rural area in North West Ethiopia [13] and the 36.7 and 7.3% rates of minimum meal frequency and minimum adequate diet respectively reported among children from the Cross-River State of Nigeria [12]. Our findings also compare favourably with the 12% of Ghanaian children meeting minimum adequate diet as reported in the 2017/2018 Multiple Indicator Cluster Survey report for Ghana [22]. Notwithstanding the differences in the prevalence rates between the current study and those of the other studies from other parts of sub-Sharan Africa, the rates of the minimum meal frequency and minimum diet diversity, minimum adequate diet and the minimum adequate diet are woefully low depicting the preponderance of poor infant and young child feeding practices in sub-Sharan Africa. It can be very difficult for mothers from poor societies such as those in Kpandai, Ghana to be able to feed their children diverse diets and the recommended number of times as they may have low income levels to be able to secure food to meet these recommendations [12].

We found in this study that about half of the mothers had adequate knowledge regarding infant and young child feeding practices, although two notable knowledge deficits were identified. Firstly, we found that, about a quarter of the mothers did not know the recommended duration for continued breastfeeding notwithstanding our finding that a greater majority of the mothers practiced continued breastfeeding. Secondly, 28% did not also know when to start appropriate complementary feeding, although all the children were receiving complementary feeding. These knowledge deficits may have negative consequences as mothers may start complementary feeding too early and may also stop continued breastfeeding before the recommended period which may not auger well for the growth and development of the child. There is thus the need for healthcare providers to emphasize these recommendations during routine nutrition education sessions of the growth monitoring promotion sessions during child welfare clinics.

Although the attitude of the mothers towards infant and young child feeding practices were positive it is important to note two important difficulties that were expressed by the majority of the mothers. We found in this study that about 52% of the mothers perceived difficulty in giving their children different types of food, although about 90% perceived this practice to be beneficial to the child. Also, a greater majority of the mothers perceived that it was beneficial to feed their children several times a day, meanwhile almost half of them perceived difficulty in doing so. Given that attitude may be a predictor of practice, these difficulties might have contributed to the low level of dietary diversity and meal frequency reported in this study. Poor income levels can also be responsible for mothers to have less desirable attitudes towards child feeding recommendations as majority of them cited lack of income as a reason for having difficulty in feeding children with diverse diets and the recommended frequencies. Individualized counselling could be provided by healthcare providers during child welfare clinics in order to identify the individual challenges and difficulties of mothers/care givers. During these sessions, mothers/care givers could be encouraged to meet the recommendations and also supported on how to overcome some of the difficulties.

Less than 30% of the children took food from both plant and animal sources of protein such as legumes and nuts, fleshy foods, dairy products and eggs. Also, the proportion of children who took Vitamin A sources of food was also low and less encouraging. These findings could be due to participants inadequate knowledge on diet diversity and consistency of diets as we found in this study that 43.5% of the mothers did not know how to diversify and enrich the diets of their children and another 72% did not know the reason why they should ensure the consistency of the diets of their children. These assertions were confirmed by our finding in this study that high level of knowledge was significantly associated with children being fed a minimum adequate diet. This finding thus demonstrate that knowledge is necessary to improving infant and young child feeding practices and justifies the need for health providers to continue to provide nutrition education to mothers during routine child welfare clinics.

Another important finding was that children whose fathers reportedly earned adequate income were several times more likely to be fed minimum adequate diet compared to their counterparts whose fathers did not earn high. This finding compares favourably with those of the 2017/2018 multiple cluster Survey report for Ghana in which minimum adequate diet and diet diversity were highest among the richest than among the poorest [22]. Obviously, fathers are the economic heads of most homes in northern Ghana and as a result are responsible for providing for the needs of the family including food. Thus, if the father earns enough income it is likely to result in the acquisition of foods to meet the complementary feeding needs of the child. There is thus the need to come out with interventions that will create nutrition awareness among fathers to enable them recognise the important role complementary feeding plays in the health and development of their children. Fathers should be encouraged to attend child welfare clinics with their spouses and be included into the nutrition education and counselling sessions.

This study is not without limitations. Its cross-sectional nature makes it difficult to establish causality. The use of a single study setting may affect the generalisability of the findings to other settings. Social desirability and recall bias may be present due to the use of a self-report instrument that required participants to recall. Notwithstanding this the study has important strengths. The findings add to the current literature regarding infant and young child feeding practices thereby increasing our understanding of the rates of infant and young child feeding practices and the associated factors. The findings provide avenues that will inform the design of interventions to improve infant and young child feeding practices in the study setting and other parts of Ghana.

## Conclusion

Mothers had notable knowledge and attitude gaps that could be addressed through nutrition education during the monthly child welfare clinics. Mother’s knowledge regarding infant and young child feeding recommendations as well as child’s father having adequate income were important determinants of the consumption of adequate diet by the children. The findings demonstrate the need for healthcare providers to continue to provide nutrition education during child welfare clinics.

## Supplementary Information

**Additional file 1.** Questionnaire used in the study.

## Data Availability

Data is available upon request from the corresponding author.

## Abbreviations

CWC: Child Welfare Clinic
ECG: Evangelical Church of Ghana
FAO: Food and Agriculture Organization
GDHS: Ghana Demographic and Health Survey
GMP: Growth monitoring and promotion
SD: Standard deviation
UNICEF: United Nations Children’s Fund
WHO: World Health Organisation
