# Supplementary material for: Mothers’ knowledge and attitudes regarding child feeding recommendations, complementary feeding practices and determinants of adequate diet
Source: BMC Nutr. 2020 Dec 1;6:67. doi: 10.1186/s40795-020-00393-0 (PMC7706028; doi:10.1186/s40795-020-00393-0)

**Questionnaire**

| **SOCIO DEMOGRAPHIC QUESTIONNAIRE FOR MOTHERS/CAREGIVERS AND**  **INFANT/YOUNG CHILDREN: ADAPTED FROM**  **WWW.FAO.ORG/DOCREP/019/I3545E/I3545E00.HTM.** | | |
| --- | --- | --- |
| **No** | **Question** | **Responses** |
| **SOCIO DEMOGRAPHIC DATA**  CAREGIVER | | |
| 1 | Age | ……………….. |
| 2 | Gender | Male ………………………………………1  Female…………………………………….2 |
| 3 | Marital status | Single……………………………………..1  Married ………………………………….2  Widowed/Separate/Divorce………………3 |
| 4 | Place of residence | ………………………………………………………………………….  ………………………………………………………………………….  …………………………………………………………………………. |
| 5 | Highest education level | No education………………………………1  Primary education ………………………...2  Junior high school ………………...………3  Senior high school ...……………………...4  Tertiary education…………………………5 |
| 6 | Ethnicity (Tribe) | Nawura……………………………………..1 |

|  |  | Komkomba……………………………………2  Nanumba………………………………….3  Akan……………………………………4  Ewe…………………………………….5  Others………………………….6 |
| --- | --- | --- |
| 7 | Religious Denomination | Christianity……………………………….1  Muslem ………………………………….2  Traditionalist …………………………….3  Others ……………………………………4 |
| **SOCIO-ECONOMIC DATA** | | |
| 8 | Occupation, Are you employed? | Yes……………………………………….1  No………………………………………..2 |
| 9 | If yes, what type of job do you do? | Farming………………………………….1  Trading………………………………….2  Tradesman……………………………….3  Public/ Civil Servant…………………….4  Others…………………………………….5 |
| 10 | If Farmer, state | Subsistent…………………………………1  Semi-Commercial…………………………2  Commercial……………………………….3 |
| 11 | If Trader/ Tradesman/Public/Civil Servant, do you have a regular cash income/ are you a salaried worker | Yes…………………………………….1  No……………………………………..2 |
| 12 | Do you receive any form of financial subvention? | Yes…………………………………….1  No ……………………………………..2 |
| 13 | If yes, in what form? | Regular ……………………………….1  Casual …………………………………2 |

| 14 | What is the Occupation of the child’s father? | Farmer…………………………………1  Trader………………………………….2  Tradesman …………………………….3  Public/Civil Servant ………………….4  Others……..……..................................5 |
| --- | --- | --- |
| 15 | Does he earn enough to buy food and essentials for all the family? | Yes…………………………………….1  No………………………………………2 |

| PRACTICES | | |
| --- | --- | --- |
| 16 | **Continued breastfeeding**  Was your child breastfed or did he or she consume breastmilk yesterday during the day or at night? | Yes……………….1  No……………….2  Don’t know/no answer……3 |
| 17 | **Minimum meal frequency**  How many times did your child eat foods, that is meals and snacks other than liquids yesterday during the day or at night? | Number of times \|___\|\|___\|  Don’t know/no answer |

**18. Dietary diversity**

Now I would like to ask you about (other) liquids or foods that your child ate yesterday during the day or at night. I am interested in whether your child had the item even if it was combined with other foods.

For example, if your child ate a millet porridge made with a mixed vegetable sauce, you should reply yes to any food I ask about that was an ingredient in the porridge or sauce.

Please do not include any food used in a small amount for seasoning or condiments (like chillies, spices, herbs or fish powder); I will ask you about those foods separately.

Yesterday during the day or at night, did your child eat:

(Read the food lists. Underline the corresponding foods consumed and tick the column Yes or No depending on whether any food item of the list was consumed. Record the number of times when relevant (Group 3)).

| Group | Food lists | No | Yes |
| --- | --- | --- | --- |
| **Group 1**: Grains, roots and tubers | Porridge, bread, rice, noodles or other foods made from grains |  |  |
|  | White potatoes, white yams, manioc, cassava or any other foods made from roots |  |  |
| **Group 2**: Legumes and nuts | Any foods made from beans, peas, lentils, nuts or seeds |  |  |
| **Group 3**:  Dairy products | Infant formula, such as [insert local examples] |  | How many times? \|___\|\|___\| |
|  | Milk, such as tinned, powdered or fresh animal milk |  | How many times? \|___\|\|___\| |
|  | Yogurt or drinking yogurt |  | How many times? \|___\|\|___\| |
|  | Cheese or other dairy products |  |  |
| **Group 4**:  Flesh foods | Liver, kidney, heart or other organ meats |  |  |
|  | Any meat, such as beef, pork, lamb, goat, chicken or duck |  |  |
|  | Fresh or dried fish, shellfish or seafood |  |  |
|  | Grubs, snails or insects |  |  |
| **Group 5**:  Eggs | Eggs |  |  |
| **Group 6**:  Vitamin A fruits and vegetables | Pumpkin, carrots, squash or sweet potatoes that are yellow or orange inside |  |  |
|  | Any dark green vegetables [insert local examples] |  |  |
|  | Ripe mangoes (fresh or dried [not green]), ripe papayas (fresh or dried), musk melon [insert other local vitamin-A-rich fruits] |  |  |
|  | Foods made with red palm oil, red palm nut or red palm nut pulp sauce |  |  |
| **Group 7**:  Other fruits and vegetables | Any other fruits or vegetables |  |  |
| **Others**  (not counted in the dietary diversity score) | Any oil, fats, or butter or foods made with any of these |  |  |
|  | Any sugary foods, such as chocolates, sweets, candies, pastries, cakes or biscuits |  |  |
|  | Condiments for flavour, such as chillies, spices, herbs or fish powder |  |  |

- The baby does not consume any food other than breastmilk

|  | **NUTRITIONAL KNOWLEGDE** |  |
| --- | --- | --- |
| 19 | **Continued breastfeeding**  How long is it recommended that a woman breastfeeds her child? | Six months or less………….1  6–11 months………….2  12–23 months………….3  24 months and more ………….4  Other………….5  Don’t know………….6 |
| 20 | **Age of start of complementary foods**  At what age should babies start eating foods in addition to breastmilk? | At six months………………1  Other………………2  Don’t know………………3 |
| 21 | **Reason for giving complementary foods at six months**  Why is it important to give foods in addition to breastmilk to babies from the age of six months? | Breastmilk alone is not sufficient (enough)/……..1  Breastmilk cannot supply all the nutrients needed for growth/from six months, baby needs more food in addition to breastmilk…………….2  Other…………….3  Don’t know…………….4 |
| 22 | **Consistency of meals**  Please look at these two pictures of porridges seen at the end of the questionnaire. Which one do you think should be given to a young child? | Shows the thick porridge…………..1  Shows the watery. …………………2  Does not know…………………….3 |
| 23 | **Reason for consistency of meals**  Why did you pick that picture? | Because the first porridge is thicker than the other………………1  Because the thick porridge is more nutritious/because it is prepared with different types of foods or ingredients (food diversity) ……………2  Other……………………..3  Don’t know……………4 |
| 24 | **Dietary diversity and ways of enriching porridge**  To feed their children, many mothers give them maize porridge.  Please tell me some ways to make maize porridge more nutritious or better for your baby’s health. | Animal-source foods (meat, poultry, fish, liver/organ meat, eggs, etc.)………………..1  Pulses and nuts: flours of groundnut and other legumes (peas, beans, lentils, etc.), sunflower seed, peanuts, soybeans …………2  Vitamin-A-rich fruits and vegetables (carrot, orange-fleshed sweet potato, yellow pumpkin, mango, papaya, etc.)……….3  Green leafy vegetables (e.g. spinach)……………..4  Energy-rich foods (e.g. oil, butter/ghee)………..5  Other…………….6  Don’t know……..7 |
| 25 | **Responsive feeding**  Do you know any ways to encourage young children to eat? | Giving them attention during meals………1  talk to them………..2  make meal times happy times………….3  clap hands………….4  make funny faces/play/laugh..5  demonstrate opening your own mouth very wide/modelling how to eat………..6  say encouraging words…..7  draw the child’s attention…8  Other……………8  Don’t know……..9 |

| ATTITUDES | | |
| --- | --- | --- |
| 26 | **Self-confidence**  How confident do you feel in preparing food for your child? | Not confident………………1  Ok/so-so……………………2  Confident……………………3  If Not good: Can you tell me the reasons why you do not feel confident?....................................... |
|  | Giving a diversity of food (foods from many food groups) |  |
| 27 | Perceived benefits How difficult is it for you to give different types of food to your child each day? | Not good…………………1  You’re not sure…………..2  Good………………………3  If Not good:………………..4  Can you tell me the reasons why it is not good?....................... |
| 28 | Perceived barriers How difficult is it for you to give different types of food to your child each day? | Not difficult……………….1  So-so……………………...2  Difficult…………………..3  If Difficult:  Can you tell me the reasons why it is difficult?.............................. |
|  | **Feeding frequently** |  |
| 29 | Perceived benefits How good do you think it is to feed your child several times each day? | Not good……………….1  You’re not sure……………….2  Good……………………3  If Not good: Can you tell me the reasons why it is not good?..................... |
|  | Perceived barriers How difficult is it for you to feed your child several times each day? | Not difficult…………...1  So-so………………….2  Difficult………………3  If Difficult: Can you tell me the reasons why it is difficult?......................... |
|  | Continuing breastfeeding beyond six months |  |
| 30 | Perceived benefits How good do you think it is to continue breastfeeding beyond six months? | Not good………………1  You’re not sure………2  Good………………3  If Not good: Can you tell me the reasons why it is not good?............................. |
| 31 | Perceived barriers How difficult is it for you to continue breastfeeding beyond six months? | Not difficult…………….1  So-so……………………2  Difficult……………3  If Difficult: Can you tell me the reasons why it is difficult?............................. |

**DATA OF CHILD**

| 32 | Age |  |
| --- | --- | --- |
| 33 | Gender | Male……………….1  Female…………….2 |
| 34 | Anthropometry | Current weight……….….  Current Length……. …..  MUAC………………… |

###

### Support material: porridges

#### 1.


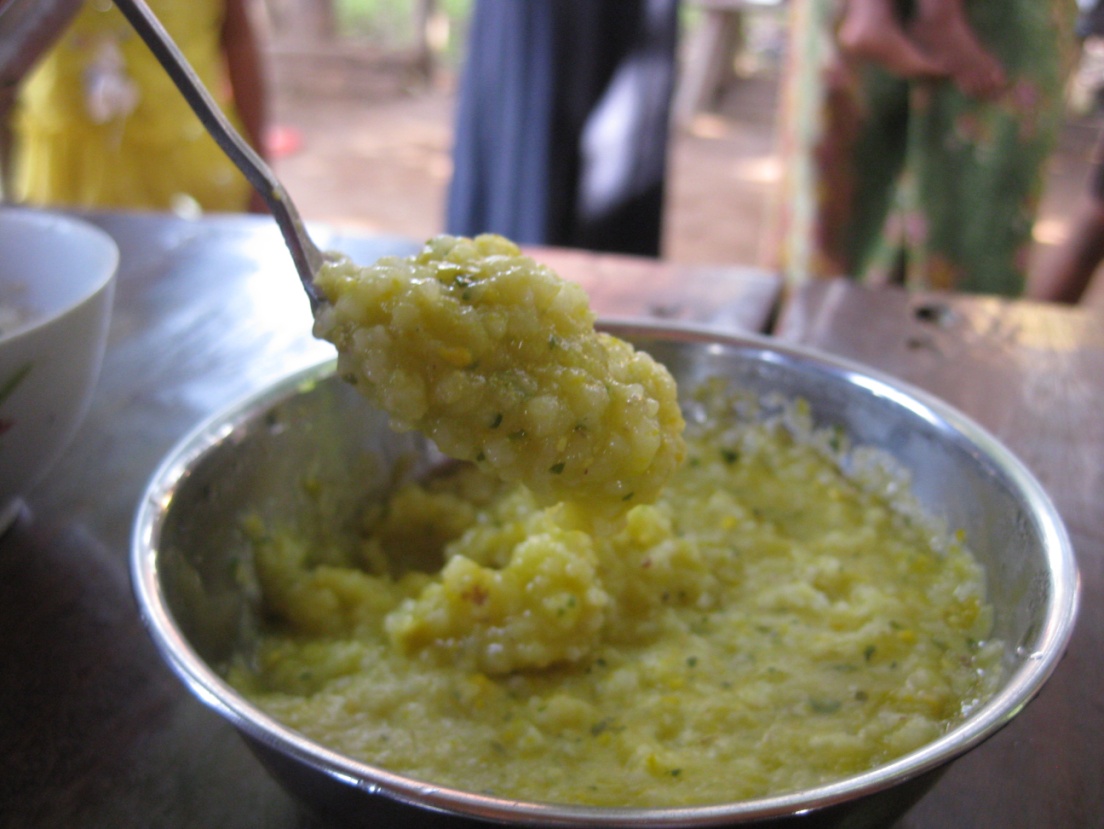


#### 2.

####
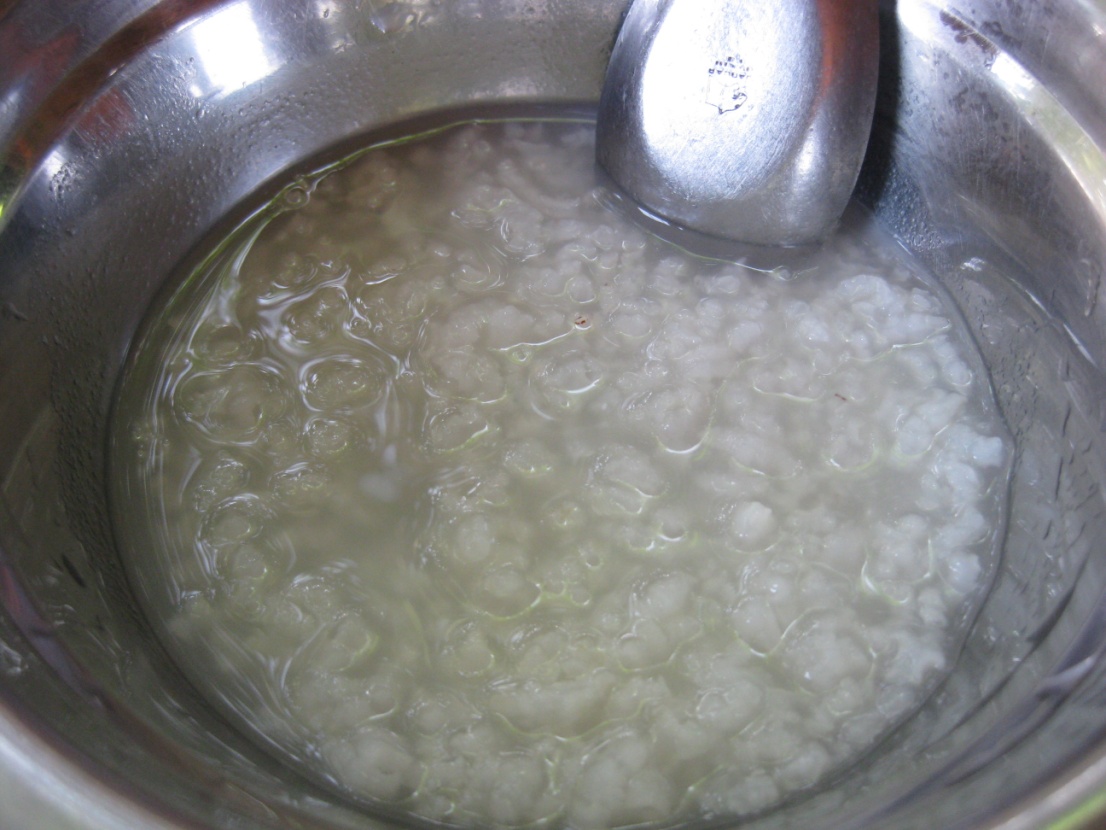

Supplement: Supplementary file 1 — Additional file 1. Questionnaire used in the study. [file 40795_2020_393_MOESM1_ESM.docx]
